# Factors Governing the Chemical Stability and NMR Parameters of Uracil Tautomers and Its 5-Halogen Derivatives

**DOI:** 10.3390/molecules25173931

**Published:** 2020-08-28

**Authors:** Kacper Rzepiela, Aneta Buczek, Teobald Kupka, Małgorzata A. Broda

**Affiliations:** Department of Physical Chemistry and Molecular Modeling, Faculty of Chemistry, University of Opole, 48 Oleska Street, 45−052 Opole, Poland; 119680@student.uni.opole.pl (K.R.); aneta.buczek@uni.opole.pl (A.B.)

**Keywords:** 5-halogenouracil (5XU), tautomers, DFT, aromaticity, NICS, HOMA, solvent stabilization

## Abstract

We report on the density functional theory (DFT) modelling of structural, energetic and NMR parameters of uracil and its derivatives (5-halogenouracil (5XU), X = F, Cl, Br and I) in vacuum and in water using the polarizable continuum model (PCM) and the solvent model density (SMD) approach. On the basis of the obtained results, we conclude that the intramolecular electrostatic interactions are the main factors governing the stability of the six tautomeric forms of uracil and 5XU. Two indices of aromaticity, the harmonic oscillator model of aromaticity (HOMA), satisfying the geometric criterion, and the nuclear independent chemical shift (NICS), were applied to evaluate the aromaticity of uracil and its derivatives in the gas phase and water. The values of these parameters showed that the most stable tautomer is the least aromatic. A good performance of newly designed xOPBE density functional in combination with both large aug-cc-pVQZ and small STO(1M)−3G basis sets for predicting chemical shifts of uracil and 5-fluorouracil in vacuum and water was observed. As a practical alternative for calculating the chemical shifts of challenging heterocyclic compounds, we also propose B3LYP calculations with small STO(1M)−3G basis set. The indirect spin–spin coupling constants predicted by B3LYP/aug-cc-pVQZ(mixed) method reproduce the experimental data for uracil and 5-fluorouracil well.

## 1. Introduction

NMR spectroscopy has been shown as an indispensable technique for the characterization of both natural products and man-made molecular systems [1,2,3,4,5,6]. The first ones are of plant or animal origin and often exist in very low concentrations. The NMR technique of natural products is generally applied, following initial physic-chemical processes of extraction leading to an increased concentration of biologically active compounds [7,8]. On the other hand, localized NMR spectroscopy in vivo is suitable to follow millimolar concentrations of metabolites in living systems [9,10]. Proteins, aminoacids and nucleic acids are common topics studied by the NMR technique [11]. The first task is to determine their structure and next to study interactions and functions. Such works are very challenging and usually supported by molecular modelling.

Theoretical prediction of the two main parameters observed in NMR spectra, chemical shifts and indirect spin–spin coupling constants, could advance the analysis of experimental spectra, as well as predict spectral parameters of new, proposed chemical structures with improved in vivo activity, or a new method of drug delivery to target tissue. In this respect, computational chemistry has been considered as a widely used theoretical tool, supporting the synthesis of potential drugs [12,13,14].

**Figure 1:**
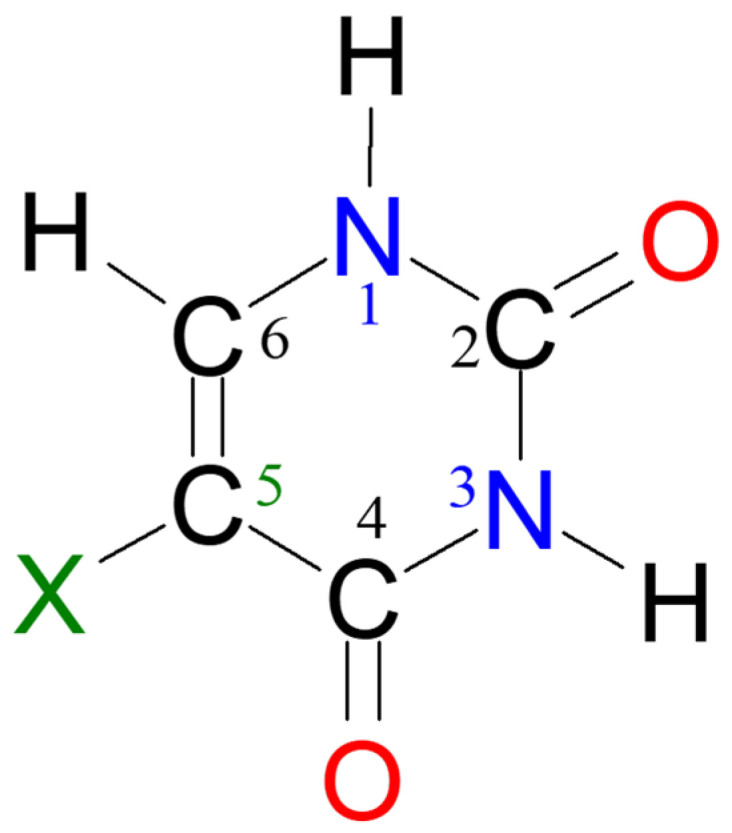
Schematic structure of uracil (U, X = H) and its four 5-halogenouracil (5XU) derivatives (X = F, Cl, Br and I) with atom numbering.

In the current study, we concentrate on uracil, a small elementary building brick of DNA—a macromolecular structure involved in “information transfer” in living systems. In this work, we will also shortly discuss its 5-halogeno derivatives (Figure 1).

Due to their mutagenic activity, the uracil analogues were prepared in the 1950s as potential antitumor drugs and applied as a powerful anti-cancer drug for more than half a century [15,16]. The 5-fluorouracil (5FU) shows a very low solubility in water (12.5 mg/L) and slightly better solubility in methanol and acetone, while DMSO is the best solvent for halogenuracils [17,18]. 5FU disturbs DNA production by inhibiting the thymidine synthesis and is administered as vein injection or as a cream in skin cancers [19]. The other 5XU have exhibited a weaker antiviral and antibacterial activity than that of 5FU and the relative order of their biological activity was F > Cl > Br > I [20]. 5ClU and 5BrU analogues are carcinogenic and 5IU has mutagenic and lethal effect on bacteriophage T4 [21].

**Figure 2:**
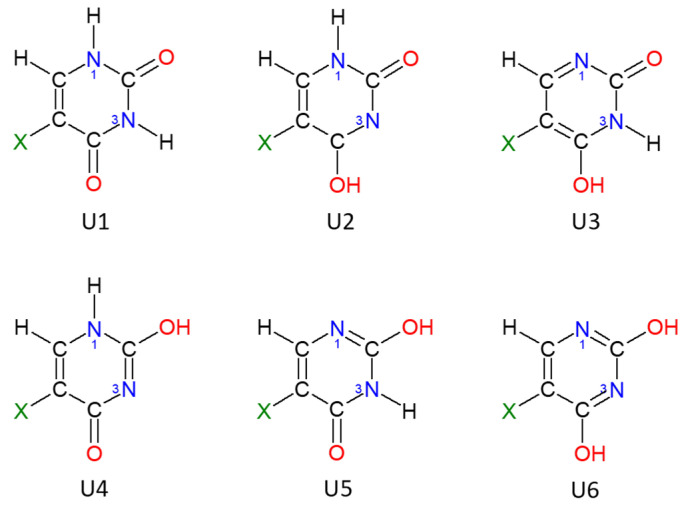
Tautomers of uracil (U, X = H) and its 5-halogen derivatives (5XU, X = F, Cl, Br or I).

Pyrimidine nitrogen bases, including uracil and its 5-halogeno derivatives, can potentially exist in several tautomeric forms (Figure 2) as free molecules [22,23,24,25] and in solution [26]. The six-membered heteroatom ring of uracil is planar, resembling aromatic molecules of benzene or pyridine. Due to its importance in nature and interesting biological activities, there have been many theoretical and experimental studies on uracil and its derivatives, for example, as reported in references [18,22,23,24,25,27,28,29,30,31,32,33]. The main scientific problems analyzed were structure [26,34,35], energetics [34,36,37], vibrational [27,28,29,30,31,38] and NMR spectra [33,39,40,41].

Surprisingly, there are only a few old studies on NMR properties of uracil available, and not all carbon–proton coupling constants in water are reported. The first proton and carbon studies were reported in early 1960s and 1970s [42,43,44]. In 1999 and 2000, more accurate multinuclear NMR studies in DMSO appeared, supported by low level ab initio prediction of chemical shifts only [39,40].

On the basis of theoretical and experimental studies, there is a general agreement that out of six possible tautomers, the diketo form (5XU1) is the most stable (Figure 2). Because of the very large energy difference between the most stable U1 structure and the remaining forms (10–20 kcal/mol), in most experiments only one tautomer is observed [22,33,39,45,46,47]. Thus, the presence of “rare” or high energy forms is very difficult to measure. IR studies in Ar-matrix suggested that the other, “rare” tautomers could not exist in concentrations above 0.1% [45,48]. Moreover, NMR [39], as well as photoluminescence measurements [49], were unable to detect any other than the diketo tautomer of uracil and its derivatives. However, for years it was speculated that perturbations in DNA replications could be due to the presence of “rare” or ionized nucleobases [50]. Unfortunately, there was no explanation about the reason for the predominant stability of the diketo tautomer.

The aim of this work is an attempt to rationalize the highest stability of the diketo form of uracil and its 5-halogeno derivatives using density functional theory to model free molecules and with implicit inclusion of the solvent effect (water). Hoping that aromaticity could be one of the factors influencing the stability of the diketo tautomer, we will also analyze the problem of aromaticity in these planar ring systems. In particular, we will try to find an answer to the following question: why is tautomer No 1, visible in most experimental studies, the lowest energy one? Thus, in an attempt to rationalize the highest stability of the diketo form in terms of aromaticity, the widely used magnetic and geometric indexes of aromaticity, e.g., nuclear independent chemical shift (NICS) [51,52] and harmonic oscillator model of aromaticity (HOMA) [53,54], will be calculated. Analysis of experimental NMR spectra of cyclic organic compounds is easier than for their modifications obtained by replacing carbon atom by a heteroatom, which significantly changes the electron density in the molecule. This problem is important for many drugs containing heterocyclic fragments in their structures or F, Cl, Br or I atoms. For such molecules, a worse agreement between experimental and theoretically predicted chemical shifts and the indirect spin–spin coupling constant is usually observed [55,56].

In order to support future experimental NMR studies on challenging heterocyclic compounds, we will check the performance of newly designed xOPBE density functional and a compact basis set STO(1M)−3G for the prediction of chemical shifts and spin–spin coupling constants (SSCC) parameters for the most stable diketo tautomer of uracil and 5FU. In addition, we will check the impact of polar solvent as well as the nature of X substituent at position C5 on 5XU tautomers’ stability and their magnetic properties (see Figure 1).

## 2. Results and Discussion

### 2.1. Energy of Uracil Tautomers and Its Derivatives

In the first step, the geometries and energies of all possible tautomers of uracil and its 5-halogen derivatives are calculated in the gas phase and in water environment, modelled by the polarizable continuum model (PCM) and the solvent model density (SMD). The obtained relative energies and dipole moments for the studied tautomers are presented in Table 1. It follows that for all studied compounds, tautomer 5XU1 clearly has the lowest energy, both in the gas phase and in water. This is in agreement with earlier reports [26,57]. The second lowest energy tautomer 5XU5 is higher by more than 10 kcal/mol. The energy order of 5XU tautomers in vacuum for most of the studied compounds is as follows:

1 < 5 < 2 < 6 < 4 < 3

and only for 5-bromouracil is the order of the two highest energy tautomers, 4 and 3, reversed. In water, the stability order of high-energy tautomers is more diverse.

Analyzing the influence of a polar solvent on the energy of uracil tautomers and its derivatives, we noticed that the solvent stabilization energy, i.e., the energy difference for a given molecule in water and in vacuum, increases with its dipole moment. To illustrate this relationship, in Figure S1, the approximately linear solvent stabilization (estimated by PCM and SMD methods) dependence on the dipole moment of uracil and its 5-halogen derivatives in vacuum is shown. The R^2^ correlation coefficient for the PCM method is 0.84, and for the SMD model it is 0.77. Solvent stabilization energies calculated by the SMD method are about 5 kcal/mol higher than the analogous values estimated by the PCM method. However, the patterns observed from Figure S1 for PCM and SMD results are similar. Thus, in the next stages of our study we limited ourselves to the first method only (some results of SMD method are included in the Supporting Material).

**Figure 3:**
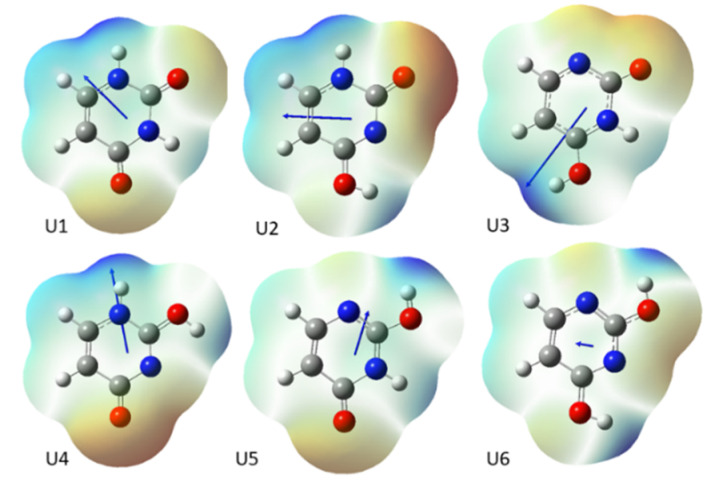
Dipole moment orientation for uracil tautomers combined with calculated maps of electrostatic potential.

In a polar environment, modelled by PCM method, the dipole moment of the studied molecules increased by 0.03 to 3.33 D. Tautomers 6 of all uracil derivatives show the lowest dipole moments in vacuum and water induces the smallest increase in their dipole moments. On the contrary, the largest calculated dipole moments are observed for tautomers 3 and 4 (see Table 1). Obviously, for these tautomers, one could notice the strongest impact of polar solvent on their dipole moments. The directions of the dipole moment vectors of uracil tautomers and the maps of electrostatic potential around these molecules are shown in Figure 3.

It is apparent from Figure 3 that the position of the exchangeable proton in all the studied tautomers is crucial and it decides on the distribution of electrostatic potential (and electron density), which is manifested by the direction and magnitude of the total dipole moment. This should control the tautomer stability, as well as the NMR parameters, which are dependent on the magnitude of magnetic field shielding by local electron density. The NMR properties of the studied tautomers will be discussed in detail in a subsequent section.

Analyzing the tautomer energies of uracil and its halogen derivatives, the following question arises: what is the reason for the high stability of tautomer 1? From Figure 3, we could speculate that this is due to the favorable configuration of two C=O and two N-H groups, which in this tautomer form three intramolecular dipole–dipole attractions due to the anti-parallel arrangement of N-H and O=C bond dipoles. In other tautomers, there is a repulsion of negative charges of free electron pairs of oxygen and nitrogen atoms lying close together.

To check whether dipole interactions are responsible for the particularly high stabilization of tautomer 1 of the studied compounds, we performed additional B3LYP-D3/aug-cc-pVQZ calculations on N-formylformamide as a model molecule. The corresponding three tautomeric forms of N-formylformamide in the gas phase are shown in Figure S2. The calculations showed that tautomer I is about 17 kcal/mol more stable than II, and 22 kcal/mol more stable than III. These results confirm that the mutual arrangement of the C=O, N-H, -OH and >N: groups in uracil molecule can change the energy of the system by about 20 kcal/mol. In addition, it can be seen that the dipole moment vectors in tautomers 1 and 2 of uracil have the same directions as in tautomers I and II of N-formylformamide, i.e., it suggests that this fragment of the molecule determines the electron density distribution in uracil.

### 2.2. Aromaticity of Uracil, 5XU and Their Tautomers

It is generally accepted that planarity and stability of benzene in comparison to other unsaturated ring compounds is due to aromaticity [58]. However, there is a long lasting controversy about aromaticity of uracil [59,60,61]. Therefore, to get more hints about the structural stability of 5XU1, we additionally considered three isomers of diazines. The calculated magnetic and geometric indexes of aromaticity for 5XU1, diazines and model aromatic compound (benzene and pyridine) are shown in Table 2. In the supporting information, the NICS and HOMA parameters for all 5XU tautomers in vacuum and water, modelled with PCM and SMD methods, are gathered in Table S1. In the case of diazines, NICS(0) indexes are smaller than for benzene, included here as the best model of an aromatic compound. However, NICS(1), NICS(1)_zz_ and HOMA values for diazines and benzene are similar, which indicates a comparable magnitude of aromaticity. Both NICS and HOMA indexes have a negligible decrease in water (Table 2).

NICS(0), NICS (1) and NICS (1)_zz_ values of tautomer **1** of uracil are negative and their absolute values are significantly smaller than for benzene, indicating its very low degree of aromaticity (Table 2). In addition, HOMA is only about 0.5, which also suggests a small aromaticity of uracil (tautomer U1). There is a controversy about the relative aromaticity of U1 and 5XU1, as apparent from data shown in Table 2. Thus, the NICS (0) and NICS (1) indexes of 5-halogeno derivatives 5XU1 are slightly more negative, indicating a negligible pronounced aromaticity, but NICS (1) _zz_ and HOMA somehow decrease, which points towards the lowering of their aromaticity. However, all indexes agree about increased aromaticity due to the presence of a polar solvent.

In the case of 5XU tautomers in the gas phase and water (see Table S1), the applied indexes of aromaticity show a significant diversity. Thus, the tautomers **1** and **4** for all compounds are the least aromatic according to NICS (0), NICS (1), NICS (1)_zz_, as well as HOMA parameters. On the contrary, tautomer **6** is characterized by large NICS indexes and its HOMA is close to unity, and therefore is considered the most aromatic one. The halogen substituent at C5 position slightly increases the ring aromaticity according to NICS indexes but lowers the HOMA parameter. Water enhances the aromaticity of all uracil tautomers except tautomer 6, for which the polar environment reduces the aromaticity measured by HOMA and NICS parameters. However, the magnitude of changes of the calculated indexes are relatively small.

### 2.3. Chemical Shifts and Indirect Spin-Spin Coupling Constants of Uracil and 5-Fluorouracil

Proton and carbon NMR spectra reflect the structural and electronic features of the studied uracil derivatives. Below we studied in detail the possibility of selected theoretical approaches to model their NMR parameters. Due to the fact that only tautomer **1** was observed in the experiment and calculated as the most stable form, we are analyzed the NMR data only for this form. Initially, we used three basis sets, STO(1M)−3G, 6−311+G (2d, p) and aug-cc-pVQZ, for the prediction of nuclear shieldings and the chemical shift of uracil and 5FU. These basis sets significantly differ by size, expressed by the number of basis functions (for example, No of b. f. = 165, 261 and 856 for 5FU) and completeness. In Tables S2 and S3, the differences between calculated and experimental chemical shifts of uracil and 5-fluorouracil tautomer **1** in vacuum and water using B3LYP functional in combination with compact STO(1M)−3G, standard 6−311+G(2d, p) and a very large aug-cc-pVQZ basis set are presented. The RMS values for carbon and proton chemical shifts, calculated with 6−311 + G (2d, p), are between those, obtained with STO(1M)−3G and aug-cc-pVQZ basis sets. The subsequent NMR calculations were conducted using a new, modified STO−3G basis set and a good quality aug-cc-pVQZ one. In order to compare the performance of xOPBE density functional with traditional B3LYP one, we conducted GIAO NMR studies in vacuum and water for uracil and 5FU (see Table 3 and Table 4).

**Figure 4:**
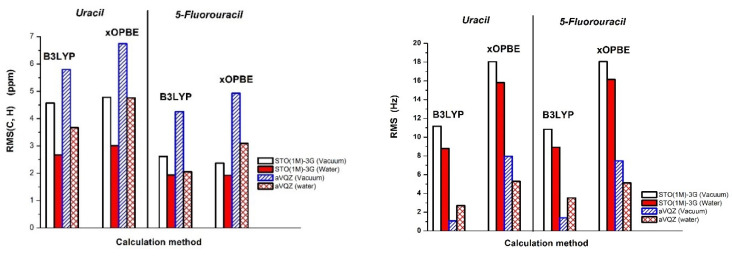
Root-mean-square deviations from experimental values of (**Left**) chemical shifts and (**Right**) indirect spin-spin coupling constants of uracil and 5-fluorouracil, calculated with selected density functional and basis set in the gas phase and water.

In the case of heterocyclic compounds, the prediction of chemical shifts could be quite challenging. As can be seen from the data collected in Table 3, both density functionals, B3LYP and xOPBE, are able to fairly accurately predict chemical shifts of uracil with RMS (C, H) below 7 and 5 ppm in vacuum and water, respectively (see Figure 4 Left). Interestingly, a small and compact STO(1M)−3G basis set, specially designed for calculation of magnetic shieldings, performs very well (RMS (C, H) of 2.7 and 3.0 ppm) in comparison to the very expensive aug-cc-pVQZ one. The importance of the inclusion of the solvent effect is also apparent from Table 3. The PCM approach, though very simplified, produces nearly two times better agreement between the predicted ^13^C and ^1^H chemical shifts and the experiment. The presence of the solvent effect also improves the quality of ^17^O chemical shifts and only xOPBE/STO(1M)−3G produces worse agreement with the experiment in water. On the contrary, for all studied theory levels, the ^15^N chemical shifts calculated in water are less accurate than for uracil in the gas phase. However, one should notice that in the case of ^17^O and ^15^N NMR, no experimental data in water are available.

As for 5-fluorouracil, theoretically predicted carbon and proton chemical shifts (Table 4) reproduce the experimental data in water very well, as evidenced by RMS (C, H) from 1.9 to 5.0 ppm. The inclusion of water via PCM significantly improves the quality of proton and carbon chemical shifts. For example, the RMS (C, H) values of isolated 5FU and in water calculated with B3LYP and xOPBE combined with STO(1M)−3G basis set decrease from 2.6 to 1.9. and from 2.4 to 1.9 ppm, respectively. As before, the small basis set STO(1M)−3G is able to accurately model 5FU shifts. Both density functionals work equally well with the modified STO−3G basis set. A surprisingly good performance of the STO(1M)−3G basis set in the prediction of heteronuclear chemical shifts, in particular in combination with B3LYP density functional, is apparent from Table 4. For example, the calculated ^19^F deviations in water are below 3 ppm.

The second most important parameter analyzed in NMR spectra is J-coupling. The so-called indirect spin–spin coupling constants (SSCC) are more difficult than nuclear shieldings to predict accurately. This is mainly due to the fact that basis sets designed to predict energy and chemical reactions are well defined for valence electrons, far from the nuclei. However, in case of SSCC, it is necessary to account for accurate description of electron density at and near the nuclei.

In Table 5, deviations from the experiment of theoretically predicted SSCC values of uracil calculated with B3LYP and xOPBE density functionals combined with small STO(1M)−3G basis set and a very large aug-cc-pVQZ basis set are presented. In both cases, we used the option “mixed” to adjust the basis set for the accurate prediction of the dominating Fermi contact term in SSCC. In the case of B3LYP/aug-cc-pVQZ calculations, all deviations from the experiment are fairly small. The RMS values are 1.1 to 2.7 Hz (without ^1^J(C5H5) results) in vacuum and water, respectively (see Figure 4 Right). B3LYP/STO(1M)−3G predicted coupling constants which are significantly less accurate and the RMS values are about 11.2 to 8.8 Hz. This is caused by a very large underestimation of one-bond C-H couplings by about 20–30 Hz. In the case of xOPBE density functional combined with both a large basis set and a small one, the predicted coupling constants are significantly worse than those obtained with B3LYP. Similarly as above, this is mainly due to one-bond C-H coupling underestimation by about 40 and 15 Hz for small and large basis sets, respectively.

In Table 6, deviations from the experiment of theoretically predicted SSCC values of 5FU calculated with B3LYP and xOPBE density functionals combined with two basis sets are presented. The presence of a fluorine atom in 5FU significantly complicates the modeling of SSCC parameters. First of all, a very large one bond coupling is visible—the experimentally observed ^1^J (C5F5) in DMSO reaches 227 Hz and for two bond carbon–fluorine couplings, the values are also high: 31 and 25 Hz for ^2^J (F5C6) and ^2^J (F5C4), respectively. The combination of B3LYP with the large basis set allows for very accurate prediction of SSCC parameters (RMS of 1.4 and 3.5 Hz in vacuum and water) and only the in case of ^1^J (C5F5) does the theory markedly overestimate the experiment (by 86 and 70 Hz). A surprisingly good performance of STO(1M)−3G basis set in prediction of ^1^J (C5F5), with deviation of 8.7 and −4.6 Hz in vacuum and water, in comparison to the Dunning-type basis set (86.1 and 69.6 Hz, respectively) is also apparent from Table 6. However, in combination with B3LYP and xOPBE density functionals, this basis set underestimates the observed ^1^J (C6H6) by about 25 and 40 Hz. The latter density functional combined with the very large basis set somehow produces a larger RMS (7.5 and 5 Hz in vacuum and water) than B3LYP.

It is apparent from the quality of the predicted SSCC parameters for uracil and 5-fluorouracil that inclusion of water could significantly improve the results. The STO(1M)−3G basis set gives larger deviations than the aug-cc-pVQZ one. However, this was expected since the former basis set was designed for good prediction of nuclear magnetic shieldings and the option “mixed” could not improve basis functions close to nuclei. In addition, the xOPBE density functional, recently designed for the prediction of nuclear shieldings, yielded somehow larger deviations from the experiment than B3LYP.

## 3. Methods

### 3.1. Computational Methods

In the current study, we try to rationalize the relative tautomer stability of uracil and its 5XU derivatives. First, we analyze the structure and energetics of six 5XU tautomers in vacuum and in water. Geometrical changes in the studied molecules due to the presence of a polar solvent should have a direct impact on the subsequently calculated NMR parameters. We pay special attention to the chemical shift of ring proton H6 and carbons C2, C4, C5 and C6 in the studied tautomers (Figure 2), as well as diagnostic one bond J-couplings (C6-H, N1-H and N3-H). To reach this goal, it is necessary to carefully select model molecules and a reliable theoretical approach. We chose a popular B3LYP hybrid density functional for the modelling of uracil and its four 5-halogen derivatives in the gas phase and in water, described by the polarized continuum model (PCM) [64] and the solvation model based on density (SMD) [65] using standard parameters in the Gaussian 16 program package [66]. To verify that the obtained structures are true energy minima, we performed harmonic frequency calculations. The known deficiencies of B3LYP [67,68,69] were corrected by the inclusion of dispersion interactions using Grimme’s GD3 term [70]. This approach should better model the intramolecular electrostatic and π-electron interactions between various structural fragments and their relative energies in vacuum and in solution. However, only very minute geometrical changes between the B3LYP and B3LYP-D3 optimized structures were observed. In order to better describe multiple bonds and lone electron pairs, we will use a fairly complete and flexible Dunning-type correlation-consistent valence basis set of quadruple–zeta quality, augmented with a diffuse function (aug-cc-pVQZ) [71]. For iodine, we tried several combinations of available basis sets. However, there were still problems with SCF convergence and optimization of 5-iodouracil tautomers, calculated with larger basis sets. Thus, all tautomers of 5IU were optimized using 6−31 + G (d) for C, N, O, H and 6−311G for I. In addition, we managed to optimize 5IU1 tautomer with two larger basis sets, aug-cc-pVQZ for C, H, N and O, and aug-cc-pVDZ-PP for I. The magnetic index of aromaticity, NICS [51,52], was used to determine the aromaticity of the studied tautomers. In addition, the geometric index of aromaticity, HOMA [53,54], was calculated. GIAO NMR calculations for tautomer 1 of uracil and 5-fluorouracil were also performed with B3LYP and xOPBE [72] density functionals. The latter functional was recently introduced for ^1^H and ^13^C chemical shift calculations. For the calculation of nuclear shieldings, we used 6−311 + G(2d,p), aug-cc-pVQZ basis sets and a relatively small and efficient STO(1M)−3G one, designed by Leszczynski et al. [73,74], for the accurate prediction of ^13^C NMR chemical shifts in large hydrocarbons.

There was no impact of GD3 term on the calculated NMR parameters and all reported nuclear shieldings and coupling constants were calculated without GD3 correction. Theoretical chemical shifts in the gas phase and water were obtained from the corresponding reference molecules, calculated at the same level of theory as nuclear shieldings of uracil and 5FU. The ^1^H and ^13^C chemical shifts were calculated with respect to benzene as a secondary reference. Similarly, ^17^O, ^15^N and ^19^F chemical shifts were calculated with respect to water, nitromethane and CFCl_3_. Following an earlier theoretical study by Alkorta and Elguero [75], describing known problems with obtaining experimental and theoretical shieldings for challenging CH_3_NO_2_ molecule, we used σ(nitromethane) = −143 ppm, as a reference for nitrogen chemical shifts.

### 3.2. NMR Experiment

To check the accuracy of previously reported experimental parameters of uracil in water, including spin–spin coupling constants, we performed several NMR experiments in D_2_O. Saturated solution of uracil (Sigma-Aldridge, Merck, Poznań, Poland, used without additional purification) was measured in 5 o. d. mm NMR tube at 20 °C using Bruker 400 Ultrashield NMR spectrometer (Karlsruhe, Germany) with DSS as a reference. Typical 1D proton and carbon spectra were recorded (carefully shimmed to obtain linewidth below 0.3 Hz). Additionally, the ^13^C (+ ^1^H) spectrum was recorded.

## 4. Conclusions

B3LYP-D3/aug-cc-pVQZ structures and energetics of uracil and its 5-halogen derivatives 5XU were theoretically modeled using the DFT approach in the gas phase and aqueous solution, introduced via PCM and SMD methods, and the stability of six possible tautomers was estimated. In agreement with previous theoretical and experimental works, the diketo tautomer **1** is the most stable one in the gas phase and in water. Considering Boltzmann populations for energies of tautomers differing by 10 kcal/mol, one could expect the presence of a “rare” one in concentrations of 10^−6^ to 10^−7^ only. Comparing the structure, energetics, and distribution of electron density and the relative orientation of total dipole moments in N-formylformamide tautomers and diazine izomers, selected as model molecules containing C=O, >N: and N-H polar groups in close proximity, we propose an interplay between intramolecular attraction and repulsion as the most important factor of tautomer I stability. This explains why this tautomer, being the lowest energy one due to intramolecular electrostatic interactions, is the most abundant one, as reflected in most experimental studies.

The obtained geometries were used for subsequent calculations of two aromaticity indexes, NICS and HOMA. The lowest aromaticity of tautomer **1** and the highest for tautomer **6** were visible from the values of NICS and HOMA indexes.

B3LYP and xOPBE functionals in combination with STO(1M)−3G and aug-cc-pVQZ basis sets were used for the calculation of isotropic nuclear magnetic shieldings and indirect spin–spin coupling constants for tautomer **1** of uracil and 5-fluorouracil in the gas phase and water. B3LYP/STO(1M)−3G fairly accurately predicted proton and carbon chemical shifts of uracil and 5-fluorouracil. Inclusion of water via the PCM approach improves the results. The corresponding RMS values for heteronuclear chemical shifts are higher. The use of a very recently designed xOPBE density functional also produces accurate chemical shifts, in particular in combination with the very large aug-cc-pVQZ basis set.

The calculation of SSCC for uracil and 5-fluorouracil using B3LYP/aug-cc-pVQZ(mixed) reproduces their experimental data very well. Only in the case of ^1^J (F5C5) are the deviations from the experiment very large (nearly close to 90 Hz). The coupling constants obtained with xOPBE/aug-cc-pVQZ (mixed) are visibly worse. Additonally, the performance of compact STO(1M)−3G basis set in prediction of uracil and 5-fluorouracil C-F, C-H, H-F and H-H couplings is less efficient than using the Dunning one.

We report on a new GIAO NMR approach leading to fast, yet accurate, calculations with a new basis set, STO(1M)−3G, which could favorably compete with a very expensive one, aug-cc-pVQZ. We also checked the performance of the recently designed xOPBE density functional for the accurate prediction of ^13^C NMR parameters. The obtained results at B3LYP/STO(1M)−3G level of theory are important for the theoretical support of NMR studies of heterocyclic ring systems which are difficult to characterize using a typical approach, combining a very simple method and small basis set, or a very large basis set and advanced method, including electron correlation effects. The proposed combination of both density functionals and compact basis set STO(1M)−3G could efficiently predict the ^1^H and ^13^C NMR spectra of heterocyclic compounds, with potentially improved biological activity, in particular in search of new anticancer and antifungal drugs. This could advance future research on computational NMR in drug design.

## Figures and Tables

**Table 1:** B3LYP-D3/aug-cc-pVQZ calculated relative energies (ΔE in kcal/mol), and dipole moment (μ in D) of uracil tautomers and its 5-halogeno derivatives in the gas phase and water using the polarizable continuum model (PCM) and the solvent model density (SMD).

	ΔE			μ		
Tautomer	Vacuum	PCM	SMD	Vacuum	PCM	SMD
U1 ^a^	0.00	0.00	0.00	4.46	6.12	6.93
U2	12.07	11.42	9.54	4.88	6.95	7.84
U3	21.19	18.27	15.24	7.17	10.26	11.62
U4	19.63	16.76	14.08	6.56	9.56	10.83
U5	11.61	14.32	12.95	3.31	4.63	5.45
U6	13.73	18.86	17.14	1.19	1.68	1.82
5FU1 ^b^	0.00	0.00	0.00	4.10	5.74	6.49
5FU2	12.90	12.61	10.57	3.60	5.27	6.01
5FU3	20.46	19.64	16.83	5.85	8.54	9.76
5FU4	17.06	14.27	11.69	7.02	10.22	11.57
5FU5	9.64	12.30	10.96	4.33	6.02	6.88
5FU6	12.46	17.93	16.04	0.60	0.63	0.68
5ClU1 ^c^	0.00	0.00	0.00	4.02	5.75	6.49
5ClU2	12.53	12.32	10.43	3.58	5.22	5.87
5ClU3	18.32	18.10	16.37	5.71	8.40	9.45
5ClU4	17.86	15.05	12.57	6.87	10.17	11.50
5ClU5	10.08	12.57	11.20	4.28	6.12	7.03
5ClU6	12.54	17.95	16.17	0.61	0.73	0.84
5BrU1 ^d^	0.00	0.00	0.00	3.97	5.74	6.32
5BrU2	12.44	12.22	10.08	3.62	5.32	5.86
5BrU3	18.02	17.90	17.05	5.77	8.51	9.22
5BrU4	17.97	15.17	12.73	6.77	10.10	11.22
5BrU5	10.18	12.65	11.20	4.20	6.04	6.80
5BrU6	12.58	17.95	15.80	0.55	0.67	0.72
5IU1 ^e^	0.00	0.00	0.00	3.91	5.36	6.11
5IU2	13.89	13.41	11.30	3.42	4.73	5.75
5IU3	20.22	20.00	16.33	5.69	7.95	9.44
5IU4	20.17	17.12	14.60	6.68	9.40	10.49
5IU5	11.04	12.99	11.83	4.29	5.87	6.35
5IU6	14.28	18.52	16.57	0.75	0.86	0.69

^a^—415.0218646 a.u.; ^b^—514.2932662 a.u.; ^c^—874.6508615 a.u.; ^d^—2988.644823 a.u.; ^e^—7333.66743424 a.u. using 6−31+G(d) for C, H, N, O and 6−311G basis set for I.

**Table 2:** B3LYP-D3/aug-cc-pVQZ values of nuclear independent chemical shift (NICS) and harmonic oscillator model of aromaticity (HOMA) parameters for the most stable tautomer 1 of uracil, its 5X-derivatives, diazines, pyridine and benzene in the gas phase and water modelled with PCM.

Molecule	Vacuum				Water			
	NICS (0)	NICS (1)	NICS (1)_zz_	HOMA	NICS (0)	NICS (1)	NICS (1) _zz_	HOMA
U1	−0.449	−1.141	−2.082	0.545	−0.852	−1.596	−3.298	0.644
5FU1	−2.354	−1.680	−2.150	0.526	−2.763	−2.101	−3.213	0.603
5ClU1	−1.324	−1.435	−1.773	0.469	−1.661	−1.821	−2.776	0.602
5BrU1	−1.071	−1.360	−1.515	0.472	−1.400	−1.744	−2.518	0.604
5IU1 ^a^	−0.732	−1.269	−1.287	0.504	−1.053	−1.653	−2.303	0.609
1,2-diazine	−4.924	−10.269	−29.170	0.975	−4.895	−10.231	−29.117	0.969
pyrimidine	−5.281	−9.781	−28.236	0.992	−5.253	−9.780	−28.252	0.991
1,4-diazine	−5.001	−10.088	−29.374	0.997	−4.962	−10.077	−29.353	0.997
pyridine	−6.579	−10.007	−29.470	0.993	−6.546	−9.999	−29.472	0.993
benzene	−7.828	−10.014	−30.041	0.991	−7.774	−10.000	−30.016	0.994

^a^ aug-cc-pVQZ basis sets for C, H, O, N atoms, and aug-cc-pVDZ-PP for I atom.

**Table 3:** Deviations from experiment of B3LYP and xOPBE calculated chemical shifts (in ppm) with STO(1M)−3G and aug-cc-pVQZ basis sets for uracil tautomer **1** in the gas phase and water ^a^. Separate RMS values for selected nuclei are shown.

		B3LYP				xOPBE			
		STO(1M)−3G		aug-cc-pVQZ		STO(1M)−3G		aug-cc-*p*VQZ	
Signal	Exp.	Vacuum	Water	Vacuum	Water	Vacuum	Water	Vacuum	Water
C2	155.93 ^b^	−4.61	−2.96	−7.26	−5.01	−4.86	−3.48	−8.95	−7.02
C4	170.30 ^b^	−7.12	−4.01	−8.81	−4.89	−8.15	−5.45	−11.15	−7.71
C5	103.79 ^b^	−1.12	−2.99	−2.96	−4.80	−0.07	−1.93	−2.16	−4.02
C6	146.26 ^b^	−7.12	−2.83	−7.83	−2.82	−6.75	−2.76	−7.89	−3.23
H5	5.79 ^b^	−0.94	−1.00	−0.62	−0.64	−1.04	−1.10	−0.66	−0.68
H6	7.53 ^b^	−0.62	−0.33	−0.93	−0.62	−0.67	−0.38	−1.00	−0.70
N1	−248.81 ^c^	17.27	24.11	19.60	27.89	8.65	15.21	11.27	19.18
N3	−221.35 ^c^	22.98	24.57	26.71	29.62	13.09	14.62	16.67	19.49
O2	252.5 ^c^	12.36	−6.83	34.50	12.78	−6.22	−23.02	21.21	2.22
O4	334 ^c^	20.71	−17.07	53.71	10.36	−2.45	−36.48	36.81	−1.95
RMS (C)		5.56	3.23	7.08	4.47	5.82	3.65	8.24	5.82
RMS (C, H)		4.57	2.67	5.80	3.67	4.78	3.01	6.74	4.76
RMS (N, O)		18.76	19.52	35.96	21.94	8.53	24.01	23.50	13.75

^a^ B3LYP-D3/aug-cc-pVQZ geometry in the gas phase and water used. Chemical shift references calculated at the same level of theory: benzene for ^13^C and ^1^H. water for ^17^O and MeNO_2_ for ^15^N. Experimental gas-to-liquid shift of −35.2 ppm for liquid water used [62]; ^b^ in D_2_O. from ref. [63]; ^c^ in DMSO. from ref. [39].

**Table 4:** Deviations from experiment of B3LYP and xOPBE calculated chemical shifts (in ppm) with STO(1M)−3G. aug-cc-pVQZ basis sets for 5FU tautomer **1** in the gas phase and water ^a^. Separate RMS values for selected nuclei are shown.

		B3LYP				xOPBE			
		STO(1M)−3G		aug-cc-pVQZ		STO(1M)−3G		aug-cc-pVQZ	
Signal	Exp.	Vacuum	Water	Vacuum	Water	Vacuum	Water	Vacuum	Water
C2	152.19 ^b^	−1.97	−0.52	−5.08	−3.11	−2.24	−1.06	−6.77	−5.11
C4	160.98 ^b^	−2.65	−0.25	−5.50	−2.41	−3.19	−1.17	−7.23	−4.58
C5	141.30 ^b^	4.57	3.17	3.48	2.24	3.47	2.03	1.46	0.17
C6	127.54 ^b^	−1.41	2.88	−4.64	0.39	−0.53	3.43	−4.44	0.22
H6	7.65 ^b^	−0.72	−0.38	−1.06	−0.69	−0.83	−0.50	−1.19	−0.84
N1	−261.06 ^c^	16.12	24.61	17.19	27.30	8.13	16.19	9.48	19.03
N3	−221.55 ^c^	22.23	23.77	26.01	28.92	12.56	14.02	16.27	19.09
O2	250 ^c^	12.91	−4.42	34.00	14.28	−5.98	−21.16	20.30	3.04
O4	321.3 ^c^	23.08	−15.25	56.15	12.76	0.22	−34.53	39.51	0.46
F	−169.31 ^d^	3.76	−2.51	−14.60	−21.87	11.59	5.39	−2.49	−9.59
RMS (C)		2.90	2.16	4.74	2.27	2.62	2.14	5.48	3.43
RMS (C, H)		2.62	1.94	4.26	2.06	2.37	1.93	4.93	3.09
RMS (N, O, F)		17.13	16.91	33.15	22.03	8.88	20.63	21.60	12.87

^a^ B3LYP-D3/aug-cc-pVQZ geometry in the gas phase and water used. Chemical shift references calculated at the same level of theory: benzene for ^13^C and ^1^H, water for ^17^O and MeNO_2_ for ^15^N. Experimental gas-to-liquid shift of −35.2 ppm for liquid water used [62]; ^b^ in D_2_O, this work; ^c^ in DMSO, from ref. [39]; ^d^ in D_2_O, from ref. [33].

**Table 5:** Deviation of B3LYP and xOPBE with STO(1M)−3G and aug-cc-pVQZ(mixed) basis sets calculated indirect spin–spin coupling constants (in Hz) for uracil in the gas phase and in water ^a^ with experimental values in D_2_O ^b^.

		B3LYP				xOPBE			
		STO(1M)−3G		aug-cc-pVQZ		STO(1M)−3G		aug-cc-pVQZ	
SSCC	Exp.	Vacuum	Water	Vacuum	Water	Vacuum	Water	Vacuum	Water
^1^J (C5H5)	177.83	−21.71	−21.00	7.21	8.06	−40.33	−39.74	−13.44	−12.76
^1^J (C6H6)	183.82	−29.47	−23.13	−0.92	6.65	−47.55	−41.64	−20.78	−13.74
^2^J (C5H6)	2.96	−0.82	−0.43	0.05	0.48	−2.14	−1.73	−1.97	−1.50
^3^J (C2H6)	9.42	−1.72	−1.46	−0.13	0.16	−2.23	−1.94	−0.75	−0.38
^3^J (C4H6)	10.54	−1.22	−1.24	0.57	0.56	−1.21	−1.26	0.67	0.61
^3^J (H5H6)	7.69	1.01	0.95	1.65	1.59	0.62	0.53	1.37	1.27
^2^J (H5C6)	3.64	0.77	0.45	2.07	1.64	−1.13	−1.40	−0.62	−0.94
^2^J (H5C4)	1.79	−0.21	0.23	0.29	0.82	−2.02	−1.58	−2.04	−1.50
RMS		12.97	11.07	2.75	3.80	22.09	20.39	8.83	6.70
RMS ^c^		11.17	8.78	1.09	2.69	18.04	15.80	7.96	5.30

^a^ B3LYP-D3/aug-cc-pVQZ geometry in the gas phase and water used; ^b^ this work; ^c^ without ^1^J (C5H5) results.

**Table 6:** Deviation of B3LYP and xOPBE with STO(1M)−3G and aug-cc-pVQZ(mixed) basis sets calculated indirect spin-spin coupling constants (in Hz) for 5-fluorouracil in the gas phase and in water ^a^ with experimental values in DMSO ^b^.

		B3LYP				xOPBE			
		STO(1M)−3G		aug-cc-pVQZ		STO(1M)−3G		aug-cc-pVQZ	
SSCC	Exp.	Vacuum	Water	Vacuum	Water	Vacuum	Water	Vacuum	Water
^1^J (C5F5)	227.0	8.74	−4.57	86.09	69.64	10.58	−1.12	88.60	73.41
^1^J (C6H6)	182.0	−26.54	−20.91	2.37	8.86	−45.06	−39.80	−18.13	−11.88
^2^J (C5H6)	4.1	0.28	0.16	0.79	0.65	1.63	1.48	2.69	2.41
^3^J (C2H6)	10.1	−2.33	−2.12	−0.74	−0.47	−2.86	−2.62	−1.39	−1.08
^3^J (C4H6)	7.3	−1.25	−1.30	−0.03	0.01	−0.97	−1.05	0.34	0.25
^3^J (F5H6)	6.0	2.09	0.87	−1.90	−0.53	4.30	3.25	−6.22	−4.86
^2^J (F5C6)	31.1	7.49	6.79	1.74	2.28	11.82	11.06	−3.01	−0.47
^2^J (F5C4)	25.6	7.12	8.18	−0.22	−1.66	9.02	9.95	−2.23	−3.50
RMS		10.60	8.50	30.46	24.84	17.30	15.11	32.09	26.39
RMS ^c^		10.84	8.92	1.39	3.53	18.05	16.15	7.47	5.13

^a^ B3LYP-D3/aug-cc-pVQZ geometry in the gas phase and water used; ^b^ from ref. [39]; ^c^ without ^1^J (C5F5) results.

## Supplementary Materials

The following are available online: Figure S1: PCM and SMD modelled gas-to-liquid energy differences for uracil and its 5XU tautomers vs. their dipole moments in vacuum, Figure S2: Three tautomers of N-formylformamide with overlapped maps of electrostatic potential and total dipole moment indicated, Table S1: Values of NICS and HOMA aromaticity indexes for uracil tautomers and its 5-halogen derivatives in the gas phase and water (PCM and SMD) calculated at B3LYP-D3/6-31+G(d,p) level of theory, Table S2: Deviations from experiment of B3LYP calculated chemical shifts (in ppm) with STO(1M)-3G, 6-311+G(2d,p) and aug-cc-pVQZ basis sets for uracil tautomer 1 in the gas phase and water ^a^. Separate RMS values for selected nuclei are shown, Table S3: Deviations from experiment of B3LYP calculated chemical shifts (in ppm) with STO(1M)-3G, 6-311+G(2d,p) and aug-cc-pVQZ basis sets for 5FU tautomer 1 in the gas phase and water ^a^. Separate RMS values for selected nuclei are shown.

## References

[1] ur-Rahman A., Choudhary M.I., ur-Rahman A., Choudhary M.I. (2015). Preface. Applications of NMR Spectroscopy.

[2] Fan T.W.M., Lane A.N. (2016). Applications of NMR spectroscopy to systems biochemistry. Prog. Nucl. Magn. Reson. Spectrosc..

[3] Mari H.S., Varras C.P., Atia tul W., Choudhary M.I., Siskos G.M., Gerothanassis P.I. (2019). Solvent-Dependent Structures of Natural Products Based on the Combined Use of DFT Calculations and ^1^H-NMR Chemical Shifts. Molecules.

[4] Cai W., Piner R.D., Stadermann F.J., Park S., Shaibat M.A., Ishii Y., Yang D., Velamakanni A., An S.J., Stoller M. (2008). Synthesis and Solid-State NMR Structural Characterization of ^13^C–Labeled Graphite Oxide. Science.

[5] Breton R.C., Reynolds W.F. (2013). Using NMR to identify and characterize natural products. Nat. Prod. Rep..

[6] Stockman B.J., Dalvit C. (2002). NMR screening techniques in drug discovery and drug design. Prog. Nucl. Mag. Res. Sp..

[7] Pauli G.F., Jaki B.U., Lankin D.C. (2005). Quantitative ^1^H NMR:  Development and Potential of a Method for Natural Products Analysis. J. Nat. Prod..

[8] Eldridge G.R., Vervoort H.C., Lee C.M., Cremin P.A., Williams C.T., Hart S.M., Goering M.G., O’Neil-Johnso M., Zeng L. (2002). High-Throughput Method for the Production and Analysis of Large Natural Product Libraries for Drug Discovery. Anal. Chem..

[9] Dong Z. (2015). Proton MRS and MRSI of the brain without water suppression. Prog. Nucl. Magn. Reson. Spectrosc..

[10] van der Graaf M. (2010). In vivo magnetic resonance spectroscopy: Basic methodology and clinical applications. Eur. Biophys. J..

[11] Bundi A., Wüthrich K. (1979). 1H-nmr parameters of the common amino acid residues measured in aqueous solutions of the linear tetrapeptides H-Gly-Gly-X-L-Ala-OH. Biopolymers.

[12] Shakibayi Far J., Ziglari A., Sayadian M., Shahriari S., Khalilimofrad M.S., Malakian F., Elsagh A., Mollaamin F. (2015). Drug Delivery and NMR Tensors Studies of Methamphetamine and Carbon-Nanotube Binding. J. Comput. Nanosci..

[13] Ramalho T.C., Pereira D.H., Thiel W. (2011). Thermal and Solvent Effects on NMR Indirect Spin–Spin Coupling Constants of a Prototypical Chagas Disease Drug. J. Phys. Chem. A.

[14] Gao H., Wei X., Liu X., Yan T. (2010). Comparison of Different Theory Models and Basis Sets in the Calculations of Structures and 13C NMR Spectra of [Pt(en) (CBDCA−O, O′)], an Analogue of the Antitumor Drug Carboplatin. J. Phys. Chem. B.

[15] Duschinsky R., Pleven E., Heidelberger C. (1957). The synthesis of 5-fluoropyrimidines. J. Am. Chem. Soc..

[16] Heidelberger C., Chaudhuri N.K., Danneberg P., Mooren D., Griesbach L., Duschinsky R., Schnitzer R.J., Pleven E., Scheiner J. (1957). Fluorinated pyrimidines, a new class of tumour-inhibitory compounds. Nature.

[17] Zorrilla-Veloz R.I., Stelzer T., López-Mejías V. (2018). Measurement and Correlation of the Solubility of 5-Fluorouracil in Pure and Binary Solvents. J. Chem. Eng. Data.

[18] Arakawa Y., Nakano M., Juni K., Arita T. (1976). Physical Properties of Pyrimidine and Purine Antimetabolites. I. The Effects of Salts and Temperature on the Solubility of 5-Fluorouracil, 1-(2-Tetrahydrofuryl)-5-fluorouracil, 6-Mercaptopurine, and Thioinosine. Chem. Pharm. Bull..

[19] Chabner B.A., Longo D.L. (2014). Harrison’s Manual of Oncology.

[20] Krenitsky T.A., Freeman G.A., Shaver S.R., Beacham L.M., Hurlbert S., Cohn N.K., Elwell L.P., Selway J.W.T. (1983). 3’-Amino-2’,3’-dideoxyribonucleosides of some pyrimidines: Synthesis and biological activities. J. Med. Chem..

[21] Patra A., Harp J., Pallan P.S., Zhao L., Abramov M., Herdewijn P., Egli M. (2013). Structure, stability and function of 5-chlorouracil modified A: U and G: U base pairs. Nucleic Acids Res..

[22] Colasurdo D.D., Pila M.N., Iglesias D.A., Laurella S.L., Ruiz D.L. (2017). Tautomerism of uracil and related compounds: A mass spectrometry study. Eur. J. Mass Spectrom..

[23] Tian S.X., Zhang C.F., Zhang Z.J., Chen X.J., Xu K.Z. (1999). How many uracil tautomers there are? Density functional studies of stability ordering of tautomers. Chem. Phys..

[24] Zhang R., Ceulemans A., Nguyen M.T. (2005). A theoretical study of uracil and its tautomers in their lowest-lying triplet state. Mol. Phys..

[25] Kua J. (2019). Exploring Free Energy Profiles of Uracil and Cytosine Reactions with Formaldehyde. J. Phys. Chem. A.

[26] Hanus M., Kabeláč M., Nachtigallová D., Hobza P. (2005). Mutagenic Properties of 5-Halogenuracils:  Correlated Quantum Chemical ab Initio Study. Biochemistry.

[27] Alcolea Palafox M., Tardajos G., Guerrero-Martínez A., Vats J.K., Joe H., Rastogi V.K. (2010). Relationships observed in the structure and spectra of uracil and its 5-substituted derivatives. Spectrochim. Acta A Mol. Biomol. Spectrosc..

[28] Muñoz Freán S., Alcolea Palafox M., Rastogi V.K. (2013). Effect of the microhydration on the tautomerism in the anticarcinogenic drug 5-fluorouracil and relationships with other 5-haloderivatives. J. Mol. Struct..

[29] Ortiz S., Alvarez-Ros M.C., Alcolea Palafox M., Rastogi V.K., Balachandran V., Rathor S.K. (2014). FT-IR and FT-Raman spectra of 6-chlorouracil: Molecular structure, tautomerism and solid state simulation. A comparison between 5-chlorouracil and 6-chlorouracil. Spectrochim. Acta A Mol. Biomol. Spectrosc..

[30] Rastogi V.K., Palafox M.A. (2011). Vibrational spectra, tautomerism and thermodynamics of anticarcinogenic drug: 5-Fluorouracil. Spectrochim. Acta Part. A Mol. Biomol. Spectrosc..

[31] Rastogi V.K., Palafox M.A., Mittal L., Peica N., Kiefer W., Lang K., Ojha S.P. (2007). FTIR and FT-Raman spectra and density functional computations of the vibrational spectra, molecular geometry and atomic charges of the biomolecule: 5-bromouracil. J. Raman Spectrosc..

[32] Almeida M.O., Barros D.A.S., Araujo S.C., Faria S.H.D.M., Maltarollo V.G., Honorio K.M. (2017). Study on molecular structure, spectroscopic properties (FTIR and UV–Vis), NBO, QTAIM, HOMO-LUMO energies and docking studies of 5-fluorouracil, a substance used to treat cancer. Spectrochim. Acta—Part A.

[33] Abdrakhimova G.S., Ovchinnikov M.Y., Lobov A.N., Spirikhin L.V., Ivanov S.P., Khursan S.L. (2014). 5-Fluorouracil solutions: NMR study of acid–base equilibrium in water and DMSO. J. Phys. Org. Chem..

[34] Alagona G., Ghio C., Monti S. (2001). Ab initio modeling of competitive drug–drug interactions: 5-fluorouracil dimers in the gas phase and in solution. Int. J. Quantum Chem..

[35] Shishkin O.V., Gorb L., Luzanov A.V., Elstner M., Suhai S., Leszczynski J. (2003). Structure and conformational flexibility of uracil: A comprehensive study of performance of the MP2, B3LYP and SCC-DFTB methods. J. Mol. Struct..

[36] Leszczynski J. (1992). Tautomerism of uracil: The final chapter? Fourth-order electron correlation contributions to the relative energies of tautomers. J. Phys. Chem..

[37] Shishkin O.V., Gorb L., Leszczynski J. (2000). Modeling of the Hydration Shell of Uracil and Thymine. Int. J. Mol. Sci..

[38] Leszczynśki J. (1991). Structure and properties of uracil and its sulfur analogs: A systematic study of basis set effects in Ab InitioSCF calculations. Int. J. Quantum Chem..

[39] Bednarek E., Dobrowolski J.C., Dobrosz-Teperek K., Kozerski L., Lewandowski W., Mazurek A.P. (2000). Theoretical and experimental ^1^H, ^13^C, ^15^N, and ^17^O NMR chemical shifts for 5-halogenouracils. J. Mol. Struct..

[40] Bednarek E., Dobrowolski J.C., Dobrosz-Teperek K., Sitkowski J., Kozerski L., Lewandowski W., Mazurek A.P. (1999). Theoretical and experimental ^1^H, ^13^C, ^15^N, and ^17^O NMR spectra of 5-nitro, 5-amino, and 5-carboxy uracils. J. Mol. Struct..

[41] Blicharska B., Kupka T. (2002). Theoretical DFT and experimental NMR studies on uracil and 5-fluorouracil. J. Mol. Struct..

[42] Kokko J.P., Mandell L., Goldstein J.H. (1962). An, N. m. r. Investigation of Proton Mobility in Substituted Uracils. J. Am. Chem. Soc..

[43] Kokko J.P., Goldstein J.H., Mandell L. (1961). A Nuclear Magnetic Resonance Investigation of Tautomerism and Substituent Effects in Some Pyrimidines and Related Nucleosides. J. Am. Chem. Soc..

[44] Jardetzky C.D., Jardetzky O. (1960). Investigation of the Structure of Purines, Pyrimidines, Ribose Nucleosides and Nucleotides by Proton Magnetic Resonance. II1. J. Am. Chem. Soc..

[45] Dobrowolski J.C., Rode J.E., Kołos R., Jamróz M.H., Bajdor K., Mazurek A.P. (2005). Ar-Matrix IR Spectra of 5-Halouracils Interpreted by Means of DFT Calculations. J. Phys. Chem. A.

[46] Cavalieri L.F., Bendich A. (1950). The Ultraviolet Absorption Spectra of Pyrimidines and Purines. J. Am. Chem. Soc..

[47] Iza N., Gil M., Morcillo J. (1988). Identification of ionic and tautomeric species of uracil by second derivative UV absorption spectroscopy. J. Mol. Struct..

[48] Ivanov A.Y., Leontiev V.S., Belous L.F., Rubin Y.V., Karachevtsev V.A. (2017). Infrared spectra of 5-fluorouracil molecules isolated in inert Ar matrices, and their films on graphene oxide at 6 K. Low Temp. Phys..

[49] Ostakhov S.S., Ovchinnikov M.Y., Masyagutova G.A., Khursan S.L. (2019). Luminescent and DFT Study of Keto–Enol Tautomers of 5-Fluorouracil and Its Derivatives in Aqueous Solutions. J. Phys. Chem. A.

[50] Singh V., Fedeles B.I., Essigmann J.M. (2015). Role of tautomerism in RNA biochemistry. RNA.

[51] Chen Z., Wannere C.S., Corminboeuf C., Puchta R., Schleyer P.v.R. (2005). Nucleus-Independent Chemical Shifts (NICS) as an Aromaticity Criterion. Chem. Rev..

[52] Schleyer P.v.R., Maerker C., Dransfeld A., Jiao H., Hommes N.J.R.v.E. (1996). Nucleus-Independent Chemical Shifts: A Simple and Efficient Aromaticity Probe. J. Am. Chem. Soc..

[53] Kruszewski J., Krygowski T.M. (1972). Definition of aromaticity basing on the harmonic oscillator model. Tetrahedron Lett..

[54] Frizzo C., Martins M. (2011). Aromaticity in heterocycles: New HOMA index parametrization. Struct. Chem..

[55] Walesa R., Kupka T., Broda M.A. (2015). Density functional theory (DFT) prediction of structural and spectroscopic parameters of cytosine using harmonic and anharmonic approximations. Struct. Chem..

[56] Kupka T., Mnich A., Broda M.A. (2019). Performance of revised STO(1M)−3G basis set for prediction of 5-fluorocytosine chemical shifts. Magn. Reson. Chem..

[57] Lukmanov T., Ivanov S.P., Khamitov E.M., Khursan S.L. (2013). Relative stability of keto-enol tautomers in 5,6-substituted uracils: Ab initio, DFT and PCM study. Comput. Chem..

[58] McMurry J. (2007). Organic Chemistry, 7th ed.

[59] Cysewski P. (2005). An ab initio study on nucleic acid bases aromaticities. J. Mol. Struct..

[60] Udagawa T. (2015). Theoretical analysis on the aromaticity of uracil: Important electronic configurations and solvent effect on the aromaticity. Chem. Phys. Let..

[61] Galvão T.L.P., Rocha I.M., Ribeiro da Silva M.D.M.C., Ribeiro da Silva M.A.V. (2013). Is Uracil Aromatic? The Enthalpies of Hydrogenation in the Gaseous and Crystalline Phases, and in Aqueous Solution, as Tools to Obtain an Answer. J. Phys. Chem. A.

[62] Makulski W., Wilczek M., Jackowski K. (2018). ^17^O and ^1^H NMR spectral parameters in isolated water molecules. Phys. Chem. Chem. Phys..

[63] Wisconsin In Biological Magnetic Resonance Data Bank. A Repository for Data from NMR Spectroscopy on Proteins, Peptides, Nucleic Acids, and Other Biomolecules. http://www.bmrb.wisc.edu/metabolomics/mol_summary/show_data.php?id=bmse000940.

[64] Tomasi J., Mennucci B., Cammi R. (2005). Quantum Mechanical Continuum Solvation Models. Chem. Rev..

[65] Marenich A.V., Cramer C.J., Truhlar D.G. (2009). Universal Solvation Model Based on Solute Electron Density and on a Continuum Model of the Solvent Defined by the Bulk Dielectric Constant and Atomic Surface Tensions. J. Phys. Chem. B.

[66] Frisch M.J., Trucks G.W., Schlegel H.B., Scuseria G.E., Robb M.A., Cheeseman J.R., Scalmani G., Barone V., Mennucci B., Petersson G.A. (2009). Gaussian 09, Revision, A.02.

[67] Becke A.D. (1988). Density-functional exchange-energy approximation with correct asymptotic behavior. Phys. Rev. A.

[68] Lee C., Yang W., Parr R.G. (1988). Development of the Colle-Salvetti Correlation-Energy Formula into a Functional of the Electron Density. Phys. Rev. B.

[69] Miehlich B., Savin A., Stoll H., Preuss H. (1989). Results obtained with the correlation-energy density functionals of Becke and Lee, Yang and Parr. Chem. Phys. Lett..

[70] Grimme S., Antony J., Ehrlich S., Krieg S. (2010). A consistent and accurate ab initio parametrization of density functional dispersion correction (dft-d) for the 94 elements H-Pu. J. Chem. Phys..

[71] Kendall R.A., Dunning T.H., Harrison R.J. (1992). Electron affinities of the first-row atoms revisited. Systematic basis sets and wave functions. J. Chem. Phys..

[72] Zhang J., Ye Q., Yin C., Wu A.-a., Xu X. (2020). xOPBE: A Specialized Functional for Accurate Prediction of ^13^C Chemical Shifts. J. Phys. Chem. A.

[73] Voronkov E., Rossikhin V., Okovytyy S., Shatckih A., Bolshakov V., Leszczynski J. (2012). Novel physically adapted STO ##−3G basis sets. Efficiency for prediction of second-order electric and magnetic properties of aromatic hydrocarbons. Int. J. Quantum Chem..

[74] Kapusta K., Voronkov E., Okovytyy S., Korobov V., Leszczynski J. (2018). Reconstruction of STO−3G Family Basis Set for the Accurate Calculation of Magnetic Properties. Russ. J. Phys. Chem. A.

[75] Alkorta I., Elguero J. (2003). GIAO Calculations of Chemical Shifts in Heterocyclic Compounds. Struct. Chem..

