# Supplementary material for: Factors Governing the Chemical Stability and NMR Parameters of Uracil Tautomers and Its 5-Halogen Derivatives
Source: Molecules. 2020 Aug 28;25(17):3931. doi: 10.3390/molecules25173931 (PMC7504704; doi:10.3390/molecules25173931)
Supplement: Supplementary file 1 [file molecules-25-03931-s001.pdf]

Supplementary material

for

Factors governing the chemical stability and NMR parameters of uracil tautomers and its 5-halogen derivatives

Kacper Rzepiela, Aneta Buczek, Teobald Kupka and Małgorzata A. Broda**

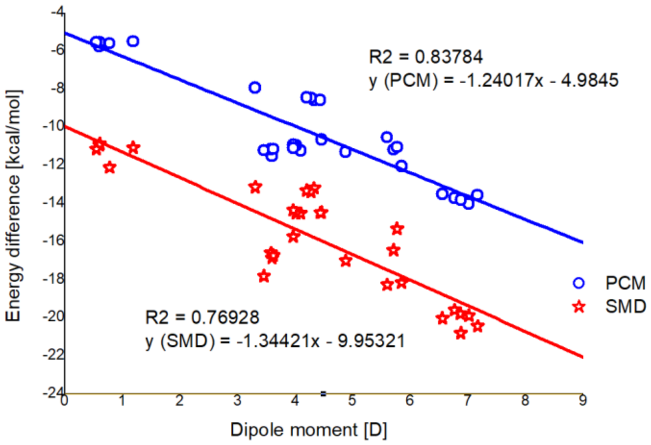

Figure S1. PCM and SMD modelled gas-to-liquid energy differences for uracil and its 5XU tautomers vs. their dipole moments in vacuum.

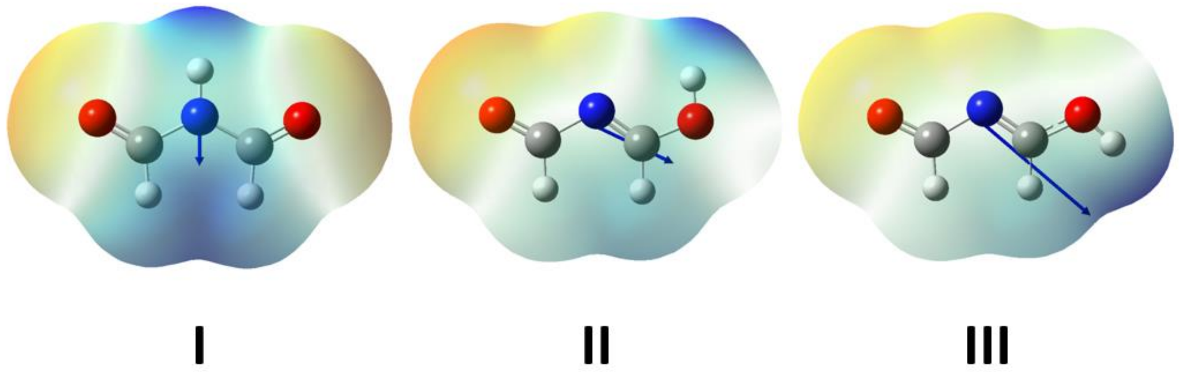

Figure S2. Three tautomers of N-formylformamide with overlapped maps of electrostatic potential and total dipole moment indicated.

Table S1. Values of NICS and HOMA aromaticity indexes for uracil tautomers and its 5-halogen derivatives in the gas phase and water (PCM and SMD) calculated at B3LYP-D3/6-31+G(d,p) level of theory

Molecule	Vacuum				water (PCM)				water (SMD)			
	NICS(0)	NICS(1)	NICS(1)zz	HOMA	NICS(0)	NICS(1)	NICS(1)zz	HOMA	NICS(0)	NICS(1)	NICS(1)zz	HOMA
U1	-0.449	-1.141	-2.082	0.545	-0.852	-1.596	-3.298	0.644	-1.266	-2.032	-4.412	0.701
U2	-1.263	-2.820	-6.695	0.727	-2.060	-3.544	-8.735	0.808	-2.462	-3.926	-9.811	0.843
U3	-1.822	-3.097	-7.421	0.772	-2.515	-3.788	-9.424	0.870	-2.759	-4.074	-10.290	0.905
U4	-0.376	-2.436	-5.803	0.445	-1.387	-3.292	-8.123	0.612	-1.880	-3.718	-9.289	0.692
U5	-1.700	-3.122	-7.867	0.675	-2.254	-3.646	-9.301	0.752	-2.526	-3.952	-10.149	0.798
U6	-5.570	-7.247	-19.420	0.983	-5.631	-7.332	-19.654	0.981	-5.369	-7.169	-19.273	0.975
5FU1	-2.354	-1.680	-2.150	0.526	-2.763	-2.101	-3.213	0.603	-3.293	-2.573	-4.363	0.664
5FU2	-3.222	-3.282	-6.568	0.698	-3.939	-3.917	-8.342	0.775	-4.437	-4.333	-9.463	0.809
5FU3	-3.353	-3.408	-6.583	0.769	-4.100	-4.066	-8.569	0.858	-4.579	-4.455	-9.685	0.893
5FU4	-2.557	-3.139	-6.559	0.439	-3.546	-3.920	-8.575	0.605	-4.116	-4.350	-9.678	0.682
5FU5	-3.752	-3.704	-8.219	0.639	-4.258	-4.161	-9.386	0.722	-4.619	-4.482	-10.206	0.769
5FU6	-7.324	-7.592	-19.080	0.970	-7.313	-7.608	-19.078	0.966	-7.168	-7.477	-18.740	0.959
5ClU1	-1.324	-1.435	-1.773	0.469	-1.661	-1.821	-2.776	0.602	-2.122	-2.242	-3.805	0.662
5ClU2	-2.189	-3.031	-6.117	0.713	-2.831	-3.634	-7.804	0.792	-3.284	-4.017	-8.828	0.828
5ClU3	-2.284	-3.092	-6.075	0.781	-2.946	-3.720	-7.925	0.872	-3.349	-4.056	-8.871	0.909
5ClU4	-1.409	-2.798	-5.793	0.382	-2.305	-3.533	-7.724	0.567	-2.824	-3.922	-8.726	0.648
5ClU5	-2.567	-3.393	-7.537	0.643	-3.032	-3.820	-8.653	0.730	-3.351	-4.105	-9.393	0.779
5ClU6	-6.097	-7.223	-18.179	0.984	-6.075	-7.235	-18.177	0.982	-5.919	-7.098	-17.812	0.978
5BrU1	-1.071	-1.360	-1.515	0.472	-1.400	-1.744	-2.518	0.604	-1.811	-2.124	-3.457	0.658
5BrU2	-1.909	-2.943	-5.813	0.717	-2.551	-3.550	-7.511	0.795	-2.965	-3.903	-8.455	0.829
5BrU3	-2.035	-2.995	-5.802	0.784	-2.679	-3.625	-7.643	0.873	-3.041	-3.928	-8.465	0.905
5BrU4	-1.157	-2.713	-5.445	0.384	-2.042	-3.439	-7.366	0.566	-2.466	-3.759	-8.210	0.639
5BrU5	-2.301	-3.314	-7.218	0.646	-2.761	-3.731	-8.326	0.731	-3.017	-3.964	-8.947	0.774
5BrU6	-5.775	-7.120	-17.787	0.985	-5.756	-7.133	-17.789	0.983	-5.562	-6.965	-17.350	0.979
5IU1	-0.732	-1.269	-1.287	0.504	-1.053	-1.653	-2.303	0.609	-	-	-	-

Table S2. Deviations from experiment of B3LYP calculated chemical shifts (in ppm) with STO(1M)-3G, 6-311+G(2d,p) and aug-cc-pVQZ basis sets for uracil tautomer **1** in the gas phase and water [a]. Separate RMS values for selected nuclei are shown

		B3LYP					
		STO(1M)-3G		6-311+G(2d,p)		aug-cc-pVQZ	
**Signal**	**Exp.**	**Vacuum**	**Water**	**Vacuum**	**Water**	**Vacuum**	**Water**
C2	155.93 [b]	-4.61	-2.96	-6.61	-4.46	-7.26	-5.01
C4	170.30 [b]	-7.12	-4.01	-8.96	-5.17	-8.81	-4.89
C5	103.79 [b]	-1.12	-2.99	-1.95	-3.76	-2.96	-4.80
C6	146.26 [b]	-7.12	-2.83	-7.78	-2.89	-7.83	-2.82
H5	5.79 [b]	-0.94	-1.00	-0.70	-0.69	-0.62	-0.64
H6	7.53 [b]	-0.62	-0.33	-1.01	-0.68	-0.93	-0.62
N1	-248.81 [c]	17.27	24.11	-9.73	-15.96	19.60	27.89
N3	-221.35 [c]	22.98	24.57	-2.62	-14.23	26.71	29.62
O2	252.5 [c]	12.36	-6.83	28.55	7.86	34.50	12.78
O4	334 [c]	20.71	-17.07	45.68	3.73	53.71	10.36
**RMS (C)**		**5.56**	**3.23**	**6.86**	**4.16**	**7.08**	**4.47**
**RMS (C,H)**		**4.57**	**2.67**	**5.62**	**3.42**	**5.80**	**3.67**
**RMS (N,O)**		**18.76**	**19.52**	**27.40**	**11.54**	**35.96**	**21.94**
No of b.f.		*152*		*240*		*824*	

[a] B3LYP-D3/aug-cc-pVQZ geometry in the gas phase and water used. Chemical shift references calculated at the same level of theory: benzene for ^{13}C and ^{1}H. water for ^{17}O and $MeNO_2$ for ^{15}N. Experimental gas-to-liquid shift of -35.2 ppm for liquid water used [1]; [b] in D_2O. from ref. [2]; [c] in DMSO. from ref. [3].

Table S3. Deviations from experiment of B3LYP calculated chemical shifts (in ppm) with STO(1M)-3G, 6-311+G(2d,p) and aug-cc-pVQZ basis sets for 5FU tautomer **1** in the gas phase and water [a]. Separate RMS values for selected nuclei are shown

		B3LYP					
		STO(1M)-3G		6-311+G(2d,p)		aug-cc-pVQZ	
**Signal**	**Exp.**	**Vacuum**	**Water**	**Vacuum**	**Water**	**Vacuum**	**Water**
C2	152.19[b]	-1.97	-0.52	-4.55	-2.67	-5.08	-3.11
C4	160.98[b]	-2.65	-0.25	-5.54	-2.55	-5.50	-2.41
C5	141.30[b]	4.57	3.17	3.72	2.48	3.48	2.24
C6	127.54[b]	-1.41	2.88	-4.20	0.74	-4.64	0.39
H5	7.65[b]	-0.72	-0.38	-16.11	-22.91	-1.06	-0.69
H6	-261.06[c]	16.12	24.61	-1.16	-0.77	17.19	27.30
N1	-221.55[c]	22.23	23.77	-14.44	-18.85	26.01	28.92
N3	250[c]	12.91	-4.42	-6.25	-17.78	34.00	14.28
O2	321.3[c]	23.08	-15.25	28.24	9.60	56.15	12.76
O4	-169.31[d]	3.76	-2.51	48.73	6.46	-14.60	-21.87
**RMS (C)**		**2.90**	**2.16**	**4.55**	**2.25**	**4.74**	**2.27**
**RMS (C,H)**		**2.62**	**1.94**	**4.10**	**2.04**	**4.26**	**2.06**
**RMS (N,O)**		**17.13**	**16.91**	**27.13**	**16.31**	**33.15**	**22.03**
No. of b.f.		*165*		*261*		*856*	

[a] B3LYP-D3/aug-cc-pVQZ geometry in the gas phase and water used. Chemical shift references calculated at the same level of theory: benzene for ^{13}C and ^{1}H. water for ^{17}O and $MeNO_2$ for ^{15}N. Experimental gas-to-liquid shift of -35.2 ppm for liquid water used [1] ; [b] in D_2O, this work; [c] in DMSO. from ref. [3]; [d] in D_2O. from ref. [4].

References

1. Makulski, W.; Wilczek, M.; Jackowski, K., 17O and 1H NMR spectral parameters in isolated water molecules. *Phys. Chem. Chem. Phys.* **2018,** 20, (35), 22468-22476.
2. Wisconsin, http://www.bmrb.wisc.edu/metabolomics/mol_summary/show_data.php?id=bmse000940 (accessed 10 July 2020. In *Biological Magnetic Resonance Data Bank. A Repository for Data from NMR Spectroscopy on Proteins, Peptides, Nucleic Acids, and other Biomolecules. National Magnetic Facility at Madison - Francisca Jofre, Mark E. Anderson, John L. Markley*, 2012.
3. Bednarek, E.; Dobrowolski, J. C.; Dobrosz-Teperek, K.; Kozerski, L.; Lewandowski, W.; Mazurek, A. P., Theoretical and experimental 1H, 13C, 15N, and 17O NMR chemical shifts for 5-halogenouracils. *J. Mol. Struct.* **2000,** 554, (2), 233-243.
4. Abdrakhimova, G. S.; Ovchinnikov, M. Y.; Lobov, A. N.; Spirikhin, L. V.; Ivanov, S. P.; Khursan, S. L., 5-Fluorouracil solutions: NMR study of acid–base equilibrium in water and DMSO. *J. Phys. Org. Chem.* **2014,** 27, (11), 876-883.
